# Observation of Emergent Dirac Physics at the Surfaces of Acoustic Higher‐Order Topological Insulators

**DOI:** 10.1002/advs.202201568

**Published:** 2022-06-26

**Authors:** Fei Meng, Zhi‐Kang Lin, Weibai Li, Peiguang Yan, Yun Zheng, Xinping Li, Jian‐Hua Jiang, Baohua Jia, Xiaodong Huang

**Affiliations:** ^1^ Hubei Key Laboratory of Roadway Bridge and Structure Engineering Wuhan University of Technology Wuhan Hubei 430070 P. R. China; ^2^ Centre of Translational Atomaterials Faculty of Science Engineering and Technology Swinburne University of Technology Hawthorn VIC 3122 Australia; ^3^ School of Physical Science and Technology and Collaborative Innovation Center of Suzhou Nano Science and Technology Soochow University Suzhou 215006 P. R. China; ^4^ Key Laboratory of Optoelectronic Devices and Systems College of Physics and Optoelectronic Engineering Shenzhen University Shenzhen 518060 P. R. China; ^5^ State Key Laboratory of Geomechanics and Geotechnical Engineering Institute of Rock and Soil Mechanics Chinese Academy of Sciences Wuhan 430071 P. R. China

**Keywords:** acoustic high‐order topological insulator, Dirac mass, hinge state, surface state, zero refractive index

## Abstract

Using 3D sonic crystals as acoustic higher‐order topological insulators (HOTIs), 2D surface states described by spin‐1 Dirac equations at the interfaces between the two sonic crystals with distinct topology but the same crystalline symmetry are discovered. It is found that the Dirac mass can be tuned by the geometry of the two sonic crystals. The sign reversal of the Dirac mass reveals a surface topological transition where the surface states exhibit zero refractive index behavior. When the surface states are gapped, 1D hinge states emerge due to the topology of the gapped surface states. The zero refractive index behavior and the emergent topological hinge states are confirmed experimentally. This study reveals a multidimensional Wannier orbital control that leads to extraordinary properties of surface states and unveils an interesting topological mechanism for the control of surface waves.

## Graphical abstract

In a 3D acoustic higher‐order topological insulator, 2D interface states between the two sonic crystals are discovered. By adjusting the geometry of the acoustic resonators, the 2D surface can transform into a zero‐index medium or open a 1D path for sound to propagate. The phenomena can be used to realize acoustic collimation, line sound source, or new acoustic sensors.

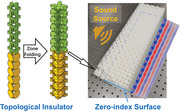

## 1 Introduction

Topological insulators (TIs) are intriguing materials which behave as insulators in the bulk but as conductors on the edges.^[^ ^1^, ^2^ ^]^ For instance, 2D TIs host topologically protected 1D edge states, while 3D TIs host 2D topological surface states. In the past years, HOTIs extend the conventional bulk‐edge correspondence to higher‐order bulk‐boundary correspondences, leading to rich multidimensional topological phenomena.^[^ ^3^, ^4^, ^5^, ^6^, ^7^, ^8^, ^9^ ^]^ For instance, a d‐dimensional HOTI can host (d‐1)‐ and (d‐2)‐dimensional boundary states simultaneously. Recently, the concept of HOTI is generalized to classical systems such as mechanical metamaterials,^[^ ^10^, ^11^, ^12^, ^13^, ^14^ ^]^ electrical circuits,^[^ ^15^, ^16^, ^17^, ^18^, ^19^, ^20^, ^21^, ^22^, ^23^, ^24^ ^]^ sonic crystals,^[^ ^25^, ^26^, ^27^, ^28^, ^29^, ^30^, ^31^, ^32^, ^33^, ^34^, ^35^ ^]^ and photonic crystals.^[^ ^36^, ^37^, ^38^, ^39^, ^40^, ^41^, ^42^, ^43^, ^44^, ^45^, ^46^, ^47^, ^48^, ^49^ ^]^ Because of their macroscopic controllability and the convenience in the excitation and detection of acoustic waves, sonic crystals stand out as a versatile platform for higher‐order topological physics.^[^ ^18^, ^19^, ^20^, ^21^, ^22^, ^23^, ^25^, ^26^, ^27^ ^]^ By designing the solid or fluid scatters/resonators in sonic crystals, we can conveniently form the desired energy bands, and the entire spectrum is easily accessible comparing to electronic systems. This allows us to realize some special manipulation of sound waves in acoustic HOTIs.

Here, we report on the experimental discovery of surface topological transitions and the emergent spin‐1 Dirac physics at the interfaces between two 3D acoustic HOTIs, which are triggered by the tunable higher‐order topology through the geometry control of the sonic crystals. Two acoustic HOTIs are created by using sonic crystals of the same crystalline symmetry but distinct higher‐order topology due to different geometry. We find that the 2D boundary states emerging at the interfaces between the two acoustic HOTIs can be described by the spin‐1 Dirac equation. The emergent surface Dirac physics is determined by the configurations of the Wannier orbitals in the two HOTIs. By tuning the geometry of two acoustic HOTIs, the Dirac mass of the 2D surface states can be tuned to experience a topological transition (i.e., a sign reversal of the Dirac mass). At the transition point, the surface states become 2D massless Dirac waves in the bulk band gap, which exhibit extraordinary properties such as zero refractive index behavior. As the 2D Dirac mass becomes finite, topological hinge states emerge due to the topology of the gapped surface states. We experimentally confirm the zero refractive index behavior and 1D sound wave propagation via topological hinge states. The findings demonstrate the rich physics and phenomena in higher‐order topological materials.

## 2 3D Acoustic HOTIs

**Figure 1:**
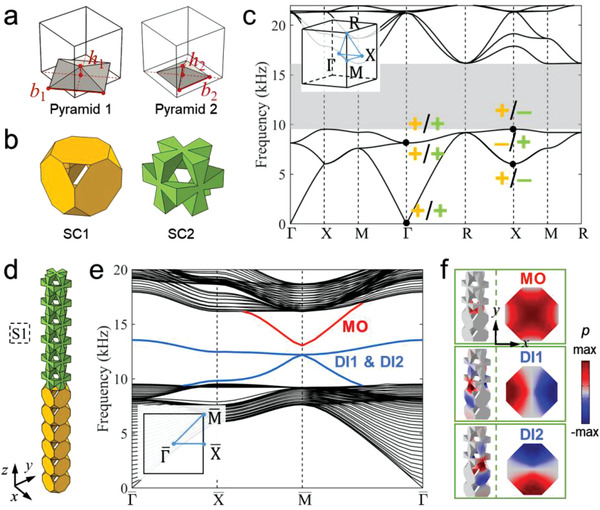
3D sonic crystals and topological surface states. a,b) Illustrations of the unit cell structures for SC1 and SC2 when *h*_1_ = *h*_2_ = 0.175*a*. c) Identical acoustic band structures of SC1 and SC2. A complete band gap appears from 9.53 to 16.13 kHz. Here, + (–) denotes even (odd) parity of the acoustic Bloch states at the Γ and *X* points (their wavefunctions are shown in Section S2, Supporting Information). Yellow (green) symbols are for SC1 (SC2). d) The supercell S1 consists of SC1 and SC2. It is finite in the *z* direction but periodic in the *x* and *y* directions. e) 2D acoustic band structure of S1. Three surface bands emerge in the bulk band gap. They are labeled as DI1, DI2, and MO according to their parity properties at the $\bar{M}$ point of the surface Brillouin zone (see the inset). f) Acoustic wavefunctions (i.e., acoustic pressure, *p*, profiles) of the surface states at the $\bar{M}$ point. Left: 3D view. Right: wavefunctions on the interface between SC1 and SC2.

We design two sonic crystals (SC1 and SC2) of the same spatial symmetry to realize different topological properties. The unit cell of SC1 has six acoustic resonators on the faces of a cubic by coupling them via the overlapped air regions. Then SC2 is realized through shifting the unit cell of SC1 by the vector (0.5*a*, 0.5*a*, 0.5*a*). *a* = 16 mm is the lattice constant. Each acoustic resonator consists of two overlapping pyramid air regions with base length *b*_1_, *b*_2_, and height *h*_1_, *h*_2_, as shown in **Figure** **1**a. In this work, *b*_1_ and *b*_2_ are fixed as 0.955*a* and 0.675*a*, respectively, while *h*_1_ and *h*_2_ varies. Figure 1b illustrates the air regions in SC1 and SC2 for *h*_1_ = *h*_2_ = 0.175*a* (see details in Section S1, Supporting Information). The sonic crystal structures are fabricated by photosensitive resin using the 3D‐printing technology. SC1 and SC2 exhibit the simple cubic lattice symmetry, that is, the space group $P_{m\bar{3}m}$.

With such constructions, SC1 and SC2 have the same acoustic band dispersion (Figure 1c). However, the symmetry properties of acoustic bands are different. In particular, the parity eigenvalues of the Bloch states at the high symmetry point X are different for SC1 and SC2, which leads to distinct topology. The difference is first characterized by the total bulk polarization of the bands below the first band gap, *P* = (*P_x_*, *P_y_*, *P_z_*). The symmetry of the simple cubic lattice guarantees *P_x_ = P_y_ = P_z_* and quantizes *P_i_* (*i* = *x*, *y*, *z*) to either 0 or 1/2. We find that *P* = (0, 0, 0) for SC2, whereas *P* = (1/2, 1/2, 1/2) for SC1 (see details in Section S2, Supporting Information).^[^ ^22^ ^]^ To fully understand the higher‐order topology, it is necessary to explore the Wannier orbitals in both acoustic HOTIs. We find that the Wannier orbitals are s‐like orbitals, yet they locate at different positions for the two sonic crystals. For SC1, the Wannier centers locate at the six surface centers of the unit cell. For SC2, the Wannier centers locate at the 12 hinge centers of the unit cell (see Section S3, Supporting Information).

To study the 2D topological surface states, we construct a ribbon‐like supercell shown in Figure 1d. The supercell (denoted as S1) is finite in the *z* direction, but periodic along the *x* and *y* directions. The calculated acoustic band structure for S1 is presented in Figure 1e. In the bulk band gap, three surface bands emerge, which can be described by the spin‐1 Dirac equation around the $\bar{M}$ point of the surface Brillouin zone, as shown in Figure 1f.

The emergence of spin‐1 Dirac physics at the interfaces between SC1 and SC2 is triggered by the topological properties of the two sonic crystals. According to topological theory,^[^ ^2^ ^]^ the Wannier centers exposed at the interface form the surface bands. There are in total three such Wannier centers located at the center and the two edge centers of the interface unit cell (see Section S3, Supporting Information). The former comes from SC1, while the latter two come from SC2. These Wannier centers form an emergent Lieb lattice at the interface leading to spin‐1 Dirac physics at the $\bar{M}$ point. We find that the Dirac mass is controlled by the energy of these Wannier orbitals. When they have the same energy, a massless spin‐1 Dirac cone emerges. In other situations, the spin‐1 Dirac mass is finite, and the surface states are gapped (see Section S4, Supporting Information).

The symmetry properties of the surface bands can be reflected by acoustic pressure fields at the $\bar{M}$ point, which is shown in the insets of Figure 1f. The acoustic wavefunctions indicate that there are one s‐like mode (labeled as MO) and two p‐like modes (labeled as DI1 and DI2) at the $\bar{M}$ point, which are consistent with the interface symmetry. Exploiting a ***k*** ***·*** ***p*** theory in the basis of the MO, DI1, and DI2 states, we find that the effective Hamiltonian for phonons around the $\bar{M}$ point is given by

(1) $$H\left(q\right)=\left[\begin{array}{ccc} 0 & iq_{x}v & iq_{y}v\\ -iq_{x}v & m & 0\\ -iq_{y}v & 0 & m \end{array}\right]+f_{0}$$

where *m* and *v* are the Dirac mass and velocity, respectively. *f*_0_ is the frequency of the Dirac point. The above analysis is also supported by an effective 2D theory based on a tight‐binding Lieb lattice model (see Section S5, Supporting Information), confirming the intriguing emergent Dirac physics at the 2D interfaces.

**Figure 2:**
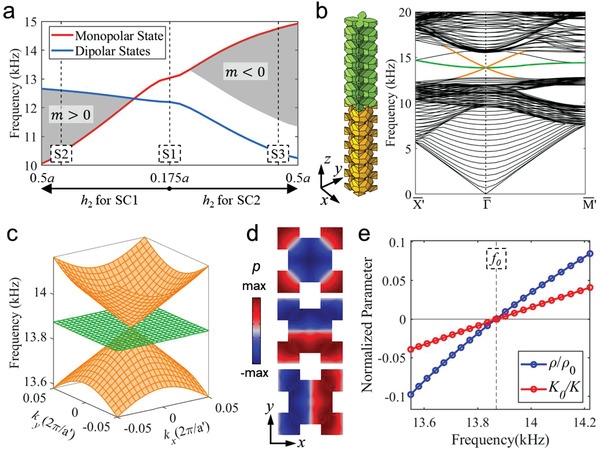
Surface topological transition. a) Frequencies of monopolar and dipolar states at the $\bar{M}$ point as a function of *h*_2_. Note that we consider the change of *h*_2_ in either SC1 (left) or SC2 (right), while other parameters remain unchanged. Gray areas denote the regions with a complete acoustic band gap for the surface states. The closing and reopening of the surface band gap accompany the sign change of the Dirac mass and the parity inversion of the surface bands at *h_2_* = 0.265a for SC1. b) The expanded supercell (left) and its band diagram (right), leading to a Dirac cone at the $\bar{\Gamma }$ point associated with the surface states in the bulk band gap. c) Conical dispersion in the vicinity of the Dirac point. $a^{\prime }=\sqrt{2}\;a$ is the lattice constant of expanded supercell in the *x*–*y* plane. d) Acoustic wavefunctions of the surface states on the interface plane between SC1 and SC2. e) The effective mass density and effective inverse bulk modulus. We use the density *ρ*_0_ and bulk modulus *K*_0_ of air to normalize the effective parameters for better graphic presentations (*ρ*_0_ = 1.21 kg m^−3^, *K*_0_ = 1.42 × 10^5^ Pa). Dashed line indicates the frequency of the Dirac point, *f*_0_= 13.87 kHz.

Calculations show that the frequencies of MO and DI states can be tuned by controlling the geometry of SC1 and SC2. The results are presented in **Figure** **2**a where we choose to keep *h*_1_ fixed and change *h*_2_ for either SC1 or SC2. Such tuning can flip the frequency order of the MO and DI states at the $\bar{M}$ point. The results indicate that the Dirac mass can be tuned to undergo a sign reversal, leading to a surface topological transition. We find that the positive Dirac mass phase is trivial and has a Wannier center at the center of the interface unit cell. The negative Dirac mass phase is topological and has two Wannier centers at the edge centers of the interface unit cell. The above picture reveals the underlying principles of the emergent Dirac physics at the surface of the HOTIs and opens a pathway for engineering the topological interface states.

## 3 Zero‐Index Conical Surface States

When the Dirac mass of the surface states vanishes, a conical dispersion emerges in the surface states at the corner of the surface Brillouin zone (i.e., the $\bar{M}$ point). Previous research^[^ ^50^ ^]^ indicates that, the accidental degenerated Dirac cone dispersion at the center of the Brillouin zone can lead to zero refractive index behavior of waves. Therefore, to trigger the zero refractive index behavior, we employ the Brillouin zone folding technique (see Section S6, Supporting Information) to bring the conical dispersion to the center of the Brillouin zone.^[^ ^34^ ^]^ It is realized by doubling the interface unit cell size to construct an expanded supercell, parameters *h*_1_ = *h*_2_ = 0.27a for SC1 and SC2. With such an expanded supercell, we create gapless surface acoustic bands (see Section S7, Supporting Information) with a spin‐1 Dirac cone at the surface Brillouin zone center (see Figure 2b–d). The Dirac point has a frequency of 13.87 kHz, which is near the middle of the bulk band gap. Protected by the bulk gap, such a surface Dirac cone with a clean dispersion creates an unprecedented realm for the manipulation of surface acoustic waves, as shown below.

**Figure 3:**
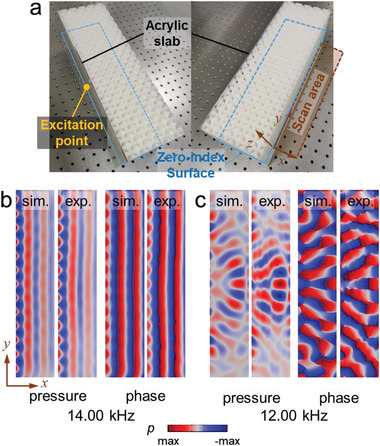
Visualization of the double‐zero‐index property of the surface Dirac cone. a) The fabricated sample and the experiment setup. The hole in the acrylic plate is used to guide acoustic waves into the system, while the detection is on the other side of the sample to detect the acoustic waves travel across the sample via the *x*–*y* interface in the middle. b,c) The simulated and the measured acoustic pressure and phase profiles in the detection *x*–*y* plane when the excitation frequency is 14.00 and 12.00 kHz, respectively.

Using the effective media theory,^[^ ^50^, ^51^, ^52^, ^53^ ^]^ we calculate the effective mass density *ρ* and the effective inverse bulk modulus 1/*K* (i.e., the compressibility) for the surface acoustic waves near the Dirac point^[^ ^35^, ^36^ ^]^ (see details in Section S8, Supporting Information). We find that these quantities undergo simultaneous sign changes at the Dirac point (Figure 2e). The simultaneous zero effective mass density *ρ* and zero effective inverse bulk modulus 1/*K* at the surface Dirac point indicate the zero refractive index property.^[^ ^50^, ^51^, ^52^, ^53^ ^]^ This property can be used to collimate the surface acoustic waves emitting from a straight edge of the interface. Our experimental setup is illustrated in **Figure** **3**a. The sample is a block with 16 × 5 expanded supercells (i.e., the supercell illustrated in Figure 2b). The left surface of the sample is glued by an acrylic slab to seal the airborne sound waves. We drill a small hole with a diameter of 5 mm in the center of the slab. The acoustic wave from the source is guided into the system via a plastic tube connected to the hole. For acoustic waves with a wavelength larger than 5 mm, the hole can be regarded as a point‐like source on the surface, which excites all possible surface states according to their frequencies. Near the surface Dirac point, such an excitation gradually merges into the excitation of the Dirac point state. The propagation of the state exhibits zero‐index feature, which can be detected by the acoustic pressure profile at the surface on the other side of the sample (see Figure 3a). As shown in Figure 3b, when the excitation frequency is 14.00 kHz, being close to the frequency of the Dirac point (*f*_0_= 13.87 kHz), both the simulated and measured acoustic pressure and phase profiles show the planar wavefront parallel to the edge of the sample. This indicates excellent surface wave collimation, even a point source is used in the experiments. Furthermore, the consistency between the simulation and the measurement confirms the wave collimation due to the surface Dirac point. In contrast, when the excitation frequency is set to 12.00 kHz (i.e., being away from the Dirac point frequency), the simulated and detected wave patterns do not show this phenomenon, which is due to the coexistence of many surface states being excited simultaneously. Such a drastic contrast supports that the surface wave collimation is caused by the zero‐index property of the surface Dirac point.

## 4 1D Hinge States Induced by Surface Topological Band Gaps

When the surface Dirac mass is finite, topological sound trapping at the 1D hinges can emerge due to the topology of the surface bands. As shown in Figure 2a, when the surface Dirac cone opens a band gap, it has either positive or negative Dirac mass. These two types of band gaps have distinct topology.^[^ ^54^, ^55^ ^]^ Protected by the fourfold rotation (*C*_4_) symmetry of the interface, the topological surface band gap has a nontrivial topological index and Wannier centers at the edges of the interface unit cell, whereas the trivial surface band gap has the vanishing topological index and the Wannier center at the interface unit cell center. When a domain wall between such two interfaces is formed, 1D topological hinge states emerge due to the surface band topology, and it is robust against imperfections around and on the hinge (Section S9, Supporting Information).

**Figure 4:**
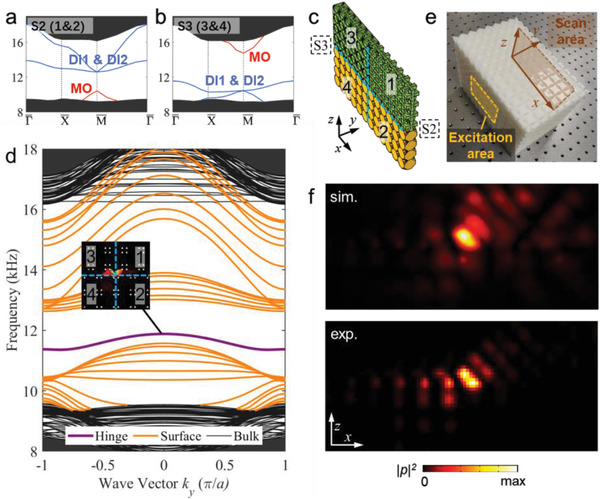
1D hinge state originates from inverted topological surface bands. a,b) Band diagrams of supercell S2 and S3 with gapped topological surface bands. c) The slab‐like hinge supercell consisted of S2 (right half) and S3 (left half). Zones 1 and 3 are the trivial sonic crystals in S2 and S3. Zones 3 and 4 are the non‐trivial sonic crystals in S2 and S3. The boundaries between zone 1 and 2 (3 and 4) are the topological interface. A hinge is formed in the center of the hinge supercell. d) Band diagram of the hinge supercell. The hinge states, surface states, and bulk states are denoted by the purple, orange, and black lines, respectively. The eigenstates around the hinge at *k_y_* = 0 (frequency 11.88 kHz) are illustrated in the inset. e) The fabricated sample and experiment setup. f) The simulated and measured sound pressure field on the scan area at 11.40 kHz.

To confirm this scenario, we use two supercells S2 (*h*_1_ = 0.175*a*, *h*2 for SC1 = 0.45*a*) and S3 (*h*_1_ = 0.175*a*, *h*2 for SC2 = 0.45*a*), as introduced in Figure 2a, to form such a domain wall. The structures of S2 and S3 are illustrated in Section S1, Supporting Information. Their acoustic band structures are presented in **Figure** **4**a,b, respectively. For S2, the surface band gap is trivial, whereas the surface band gap for S3 is topological.

We build a slab‐like supercell consisting of six S2 supercells and six S3 supercells to reveal the hinge states (see Figure 4c). We denote the two sonic crystals comprising the S2 supercell as 1 and 2, and the two sonic crystals comprising the S3 supercell as 3 and 4. The slab‐like supercell is periodic in the *y* direction, but finite in both the *x* and *z* directions. A 1D hinge boundary along the *y* direction is shared by the 1, 2, 3, and 4 regions. Due to the distinct interface states at the (1, 2) and (3, 4) interfaces, topological boundary states emerge at the 1D hinge. As shown by the acoustic band structure in Figure 4d, there are surface states and hinge states in the bulk band gap. In particular, the hinge states emerge in the surface band gap, confirming the above picture of surface topology driven hinge states.

To verify these results in experiments, we fabricate a sample consisting of eight layers of the slab‐like supercell (see Figure 4e). A speaker is used to generate sound waves impinging on the left side of the sample through a square tube (side length 5*a*). The sound pressure field at the *x–z* plane 1 mm away from the right surface is scanned. At the excitation frequency of 11.40 kHz, the simulated and measured acoustic pressure profiles are shown in Figure 4f. Both acoustic pressure profiles consistently show the emergence of hinge localized states. At frequencies higher or lower (such as 13.30 and 10.80 kHz), sound waves propagate through the sample two‐dimensionally via the topological surface states of supercell S2 or S3 (see the Experimental Results in Section S10, Supporting Information). These results confirm the emergence of the topological hinge states due to the distinct topology of the positive and negative Dirac mass surface states.

## 5 Conclusion

In this research, we design specifically a 3D acoustic higher‐order TI with three topological surface bands. The 2D topological surface states can be described by the spin‐1 Dirac equation. The surface bands and the corresponding Dirac mass can be tuned by changing the geometry of the sonic resonators. Accompanying the closing and reopening of surface band gap, there is a sign reversal of the Dirac mass and hence the topological transition. At the transition point, the surface Dirac physics emerges in the bulk band gap, and the surface states become 2D massless Dirac waves. The simultaneous zero effective mass density and infinite bulk modulus at the Dirac point are experimentally validated. Topological hinge states emerge when the 2D Dirac mass of the gapped surface states changes from positive to negative. 1D sound wave propagation via the topological hinge states and the dimensional change of sound waves are realized and experimentally verified. Our findings reveal the intriguing Dirac physics in higher‐order topological materials, which can lead to practical applications to manipulate acoustic waves.

## Conflict of Interest

The authors declare no conflict of interest.

## Supporting information

Supporting Information

## Data Availability

The data that support the findings of this study are available from the corresponding author upon reasonable request.

## References

[1] M. Z. Hasan , C. L. Kane , Rev. Mod. Phys. 2010, 82, 3045.

[2] X.‐L. Qi , S.‐C. Zhang , Rev. Mod. Phys. 2011, 83, 1057.

[3] W. A. Benalcazar , B. A. Bernevig , T. L. Hughes , Science 2017, 357, 61. 28684520 10.1126/science.aah6442

[4] J. Langbehn , Y. Peng , L. Trifunovic , F. von Oppen , P. W. Brouwer , Phys. Rev. Lett. 2017, 119, 246401. 29286744 10.1103/PhysRevLett.119.246401

[5] Z. Song , Z. Fang , C. Fang , Phys. Rev. Lett. 2017, 119, 246402. 29286745 10.1103/PhysRevLett.119.246402

[6] W. A. Benalcazar , B. A. Bernevig , T. L. Hughes , Phys. Rev. B 2017, 96, 245115.

[7] F. Schindler , A. M. Cook , M. G. Vergniory , Z. Wang , S. S. P. Parkin , B. A. Bernevig , T. Neupert , Sci. Adv. 2018, 4, eaat0346. 29869644 10.1126/sciadv.aat0346 PMC5983919

[8] M. Ezawa , Phys. Rev. Lett. 2018, 120, 026801. 29376716 10.1103/PhysRevLett.120.026801

[9] B. Su , Y. Huang , Y. H. Hou , J. Li , R. Yang , Y. Ma , Y. Yang , G. Zhang , X. Zhou , J. Luo , Z.‐G. Chen , Adv. Sci. 2022, 9, 2101532. 10.1002/advs.202101532 PMC8844473 34923770

[10] M. Serra‐Garcia , V. Peri , R. Susstrunk , O. R. Bilal , T. Larsen , L. G. Villanueva , S. D. Huber , Nature 2018, 555, 342. 29334685 10.1038/nature25156

[11] H. Fan , B. Xia , L. Tong , S. Zheng , D. Yu , Phys. Rev. Lett. 2019, 122, 204301. 31172787 10.1103/PhysRevLett.122.204301

[12] S.‐y. Huo , H.‐b. Huang , L.‐y. Feng , J.‐j. Chen , Appl. Phys. Express 2019, 12, 094003.

[13] Y. Wu , M. Yan , Z.‐K. Lin , H.‐X. Wang , F. Li , J.‐H. Jiang , Sci. Bull. 2021, 66, 1959. 10.1016/j.scib.2021.06.024 36654165

[14] C.‐W. Chen , R. Chaunsali , J. Christensen , G. Theocharis , J. Yang , Commun. Mater. 2021, 2, 62.

[15] C. W. Peterson , W. A. Benalcazar , T. L. Hughes , G. Bahl , Nature 2018, 555, 346. 29542690 10.1038/nature25777

[16] S. Imhof , C. Berger , F. Bayer , J. Brehm , L W. Molenkamp , T. Kiessling , F. Schindler , C. H. Lee , M. Greiter , T. Neupert , R. Thomale , Nat. Phys. 2018, 14, 925.

[17] M. Ezawa , Phys. Rev. B 2018, 97, 155305.

[18] J. Bao , D. Zou , W. Zhang , W. He , H. Sun , X. Zhang , Phys. Rev. B 2019, 100, 201406.

[19] S. Liu , S. Ma , Q. Zhang , L. Zhang , C. Yang , O. You , W. Gao , Y. Xiang , T. J. Cui , S. Zhang , Light Sci. Appl. 2020, 9, 145. 32864120 10.1038/s41377-020-00381-w PMC7438484

[20] W. Zhang , D. Zou , J. Bao , W. He , Q. Pei , H. Sun , X. Zhang , Phys. Rev. B 2020, 102, 100102.

[21] C. W. Peterson , T. Li , W. A. Benalcazar , T. L. Hughes , G. Bahl , Science 2020, 368, 1114. 32499440 10.1126/science.aba7604

[22] C. W. Peterson , T. Li , W. Jiang , T. L. Hughes , G. Bahl , Nature 2021, 589, 376. 33473226 10.1038/s41586-020-03117-3

[23] W. Zhang , D. Zou , Q. Pei , W. He , J. Bao , H. Sun , X. Zhang , Phys. Rev. Lett. 2021, 126, 146802. 33891442 10.1103/PhysRevLett.126.146802

[24] B. Lv , R. Chen , R. Li , C. Guan , B. Zhou , G. Dong , C. Zhao , Y. C. Li , Y. Wang , H. Tao , J. Shi , D.‐H. Xu , Communications Physics 2021, 4, 108.

[25] X. Zhang , H.‐X. Wang , Z.‐K. Lin , Y. Tian , B. Xie , M.‐H. Lu , Y.‐F. Chen , J.‐H. Jiang , Nat. Phys. 2019, 15, 582.

[26] H. Xue , Y. Yang , F. Gao , Y. Chong , B. Zhang , Nat. Mater. 2019, 18, 108. 30598539 10.1038/s41563-018-0251-x

[27] X. Ni , M. Weiner , A. Alu , A. B. Khanikaev , Nat. Mater. 2019, 18, 113. 30598540 10.1038/s41563-018-0252-9

[28] X. Zhang , B. Y. Xie , H. F. Wang , X. Xu , Y. Tian , J. H. Jiang , M. H. Lu , Y. F. Chen , Nat. Commun. 2019, 10, 5331. 31767849 10.1038/s41467-019-13333-9 PMC6877633

[29] H. Xue , Y. Yang , G. Liu , F. Gao , Y. Chong , B. Zhang , Phys. Rev. Lett. 2019, 122, 244301. 31322389 10.1103/PhysRevLett.122.244301

[30] X. Zhang , Z. K. Lin , H. X. Wang , Z. Xiong , Y. Tian , M. H. Lu , Y. F. Chen , J. H. Jiang , Nat. Commun. 2020, 11, 65. 31900420 10.1038/s41467-019-13861-4 PMC6941978

[31] M. Weiner , X. Ni , M. Li , A. Alu , A. B. Khanikaev , Sci. Adv. 2020, 6, eaay4166. 32258398 10.1126/sciadv.aay4166 PMC7101231

[32] F. Meng , Y. Chen , W. Li , B. Jia , X. Huang , Appl. Phys. Lett. 2020, 117, 151903.

[33] Y. Qi , C. Qiu , M. Xiao , H. He , M. Ke , Z. Liu , Phys. Rev. Lett. 2020, 124, 206601. 32501055 10.1103/PhysRevLett.124.206601

[34] X. Ni , M. Li , M. Weiner , A. Alu , A. B. Khanikaev , Nat. Commun. 2020, 11, 2108. 32355274 10.1038/s41467-020-15705-y PMC7193630

[35] H. Xue , Y. Ge , H.‐X. Sun , Q. Wang , D. Jia , Y.‐J. Guan , S.‐Q. Yuan , Y. Chong , B. Zhang , Nat. Commun. 2020, 11, 2442. 32415220 10.1038/s41467-020-16350-1 PMC7229046

[36] J. Noh , W. A. Benalcazar , S. Huang , M. J. Collins , K. P. Chen , T. L. Hughes , M. C. Rechtsman , Nat. Photonics 2018, 12, 408.

[37] Y. Ota , F. Liu , R. Katsumi , K. Watanabe , K. Wakabayashi , Y. Arakawa , S. Iwamoto , Optica 2019, 6, 786.

[38] X.‐D. Chen , W.‐M. Deng , F.‐L. Shi , F.‐L. Zhao , M. Chen , J.‐W. Dong , Phys. Rev. Lett. 2019, 122, 233902. 31298874 10.1103/PhysRevLett.122.233902

[39] B.‐Y. Xie , G.‐X. Su , H.‐F. Wang , H. Su , X.‐P. Shen , P. Zhan , M.‐H. Lu , Z.‐L. Wang , Y.‐F. Chen , Phys. Rev. Lett. 2019, 122, 233903. 31298912 10.1103/PhysRevLett.122.233903

[40] S. Mittal , V. V. Orre , G. Zhu , M. A. Gorlach , A. Poddubny , M. Hafezi , Nat. Photonics 2019, 13, 692.

[41] A. El Hassan , F. K. Kunst , A. Moritz , G. Andler , E. J. Bergholtz , M. Bourennane , Nat. Photonics 2019, 13, 697.

[42] M. Li , D. Zhirihin , M. Gorlach , X. Ni , D. Filonov , A. Slobozhanyuk , A. Alù , A. B. Khanikaev , Nat. Photonics 2019, 14, 89.

[43] X. Zhou , Z. K. Lin , W. Lu , Y. Lai , B. Hou , J. H. Jiang , Laser Photonics Rev. 2020, 14, 2000010.

[44] Y. T. Yang , Z. Y. Jia , Y. J. Wu , R. C. Xiao , Z. H. Hang , H. Jiang , X. C. Xie , Sci. Bull. 2020, 65, 531. 10.1016/j.scib.2020.01.024 36659184

[45] W. Zhang , X. Xie , H. Hao , J. Dang , S. Xiao , S. Shi , H. Ni , Z. Niu , C. Wang , K. Jin , X. Zhang , X. Xu , Light Sci. Appl. 2020, 9, 109. 32637076 10.1038/s41377-020-00352-1 PMC7324580

[46] H.‐R. Kim , M.‐S. Hwang , D. Smirnova , K.‐Y. Jeong , Y. Kivshar , H.‐G. Park , Nat. Commun. 2020, 11, 5758. 33188209 10.1038/s41467-020-19609-9 PMC7666194

[47] M. Kim , Z. Jacob , J. Rho , Light: Sci. Appl. 2020, 9, 130. 32704363 10.1038/s41377-020-0331-y PMC7371865

[48] L. Zhang , Y. Yang , Z.‐K. Lin , P. Qin , Q. Chen , F. Gao , E. Li , J.‐H. Jiang , B. Zhang , H. Chen , Adv. Sci. 2020, 7, 1902724. 10.1002/advs.201902724 PMC7080542 32195092

[49] Y. Liu , S. Leung , F.‐F. Li , Z.‐K. Lin , X. Tao , Y. Poo , J.‐H. Jiang , Nature 2021, 589, 381. 33473227 10.1038/s41586-020-03125-3

[50] X. Huang , Y. Lai , Z. H. Hang , H. Zheng , C. T. Chan , Nat. Mater. 2011, 10, 582. 21623377 10.1038/nmat3030

[51] C. Liu , J. Luo , Y. Lai , Phys. Rev. Mater. 2018, 2, 045201.

[52] C. Xu , G. Ma , Z. G. Chen , J. Luo , J. Shi , Y. Lai , Y. Wu , Phys. Rev. Lett. 2020, 124, 074501. 32142328 10.1103/PhysRevLett.124.074501

[53] A. M. Mahmoud , I. Liberal , N. Engheta , Opt. Mater. Express 2017, 7, 415.

[54] M. Xiao , G. Ma , Z. Yang , P. Sheng , Z. Q. Zhang , C. T. Chan , Nat. Phys. 2015, 11, 240.

[55] Y. Chen , F. Meng , G. Li , X. Huang , Acta Mater. 2019, 164, 377.

