# Supplementary material for: Observation of Emergent Dirac Physics at the Surfaces of Acoustic Higher‐Order Topological Insulators
Source: Adv Sci (Weinh). 2022 Jun 26;9(24):2201568. doi: 10.1002/advs.202201568 (PMC9404400; doi:10.1002/advs.202201568)
Supplement: Supplementary file 1 — Supporting Information [file ADVS-9-2201568-s001.pdf]

ADVANCED SCIENCE
Open Access

Supporting Information

for *Adv. Sci.*, DOI 10.1002/advs.202201568

Observation of Emergent Dirac Physics at the Surfaces of Acoustic Higher-Order Topological Insulators

Fei Meng, Zhi-Kang Lin, Weibai Li, Peiguang Yan, Yun Zheng, Xinping Li, Jian-Hua Jiang, Baohua Jia* and Xiaodong Huang**

1. Structure design of the unit cells and supercells

The two sonic crystals include a topologically non-trivial sonic crystal SC1 and a topologically trivial sonic crystal SC2. SC1 is firstly constructed by some connected air cavities, which are respectively located at each face of a cube and coupled through their overlapped regions. Then SC2 is realized through shifting the unit cell of SC1 by a vector of $\Delta \boldsymbol{r} = (0.5a, 0.5a, 0.5a)$ and shows different topological properties from SC1.

The shape of the air cavities at each face is designed as the superposition of 2 pyramid structures, labelled as pyramid 1 and pyramid 2. Their positions are illustrated in Fig. 1a in the main text. Pyramid 1 (2) has a base length of b_1 (b_2) and a height of h_1 (h_2). In this research, b_1 and b_2 are taken as $0.955a$ and $0.675a$, respectively, while h_1 and h_2 vary. Note that if $h_1 \geq h_2$, pyramid 1 covers pyramid 2 completely.

When $h_1 = h_2 = 0.175a$, we can obtain SC1 and SC2 depicted in Fig.1b in the main text, and the supercell S1 can be composed from them following the process in Fig. S1. By increasing h_2 for SC1 or SC2 to $0.45a$, we can obtain the modified sonic crystals SC1* or SC2* with bigger air cavities. Please note that their topological properties remain unchanged. Then, as displayed in Fig. S2, combining SC1* with SC2, we can obtain the supercell S2; combining SC2* with SC1, we obtain the supercell S3.

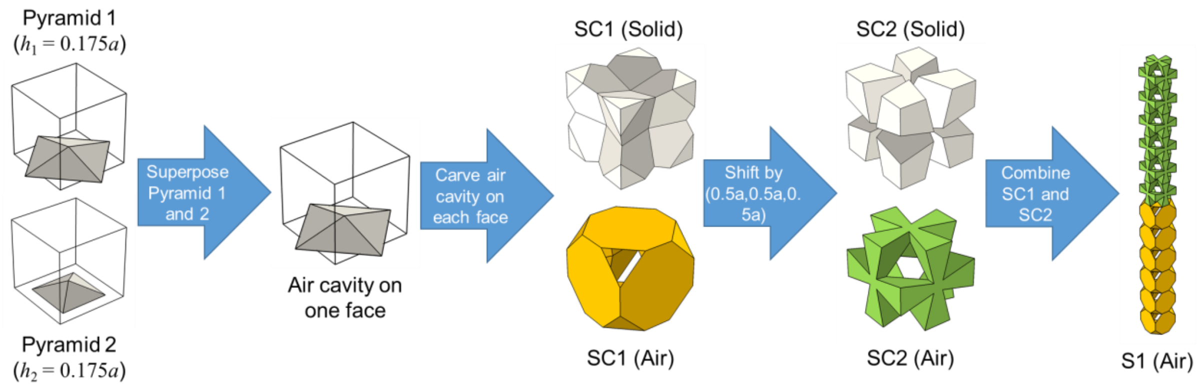

Fig. S1 | Structure design of the unit cells and supercell S1.

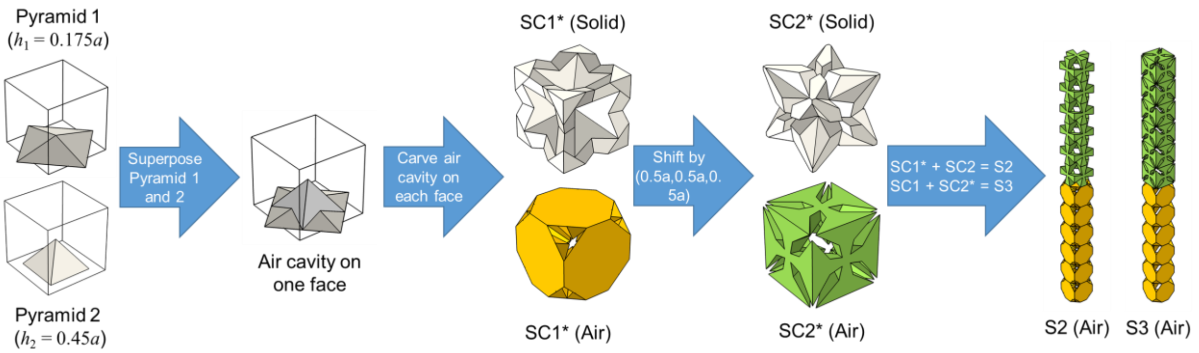

Fig. S2 | Structure design of the unit cells and supercells S2 and S3.

2. Fractional bulk polarization

For the two sonic crystals, Berry curvature vanishes everywhere in the first Brillouin zone (BZ) [1,2] due to the simultaneous restrictions of inversion symmetry of the simple cubic lattice and the time-reversal symmetry. The topological properties of these sonic crystals can instead be characterized by the fractional bulk polarization, $\mathbf{P}$ (P_x, P_y, P_z). It is the integration of the Berry connection over the momentum space [1,3],

$$\mathbf{P} = -\frac{1}{(2\pi)^3} \iiint dk_x dk_y dk_z \mathrm{Tr}[\mathbf{A}_n(\mathbf{k})], \tag{S1}$$

where $\mathbf{A}_n(\mathbf{k}) = i\langle u_n(\mathbf{k})|\partial_{\mathbf{k}}|u_n(\mathbf{k})\rangle$ is the Berry connection, $\mathbf{k} = (k_x, k_y, k_z)$ is the wavevector. n refers to the band index which runs over all the bands below the band gap. $\partial_{\mathbf{k}}$ is the vector gradient operator in $\mathbf{k}$-space. $|u_n(\mathbf{k})\rangle$ is the periodic part of the Bloch wave function. The integration in Eq. S1 is conducted over the first BZ. Due to the crystalline symmetry of the space group $P_{\mathrm{m}\bar{3}\mathrm{m}}$ (including three mirror symmetries and threefold rotation symmetry along [1,1,1]), we have $P_x = P_y = P_z$, and P_i is quantized to either 0 or $1/2$. The quantized value can be determined by the parities of the Bloch eigenstates at the high-symmetry points (HSPs) in the BZ [1,3]:

$$P_i = \frac{1}{2}\left(\sum_n q_i^n \bmod 2\right), \ (-1)^{q_i^n} = \frac{\eta_n(\mathrm{X}_i)}{\eta_n(\Gamma)}, \tag{S2}$$

The summation is taken over all the bands below the band gap. $i = x, y, z$ represents the direction. $\eta_n(\mathrm{X}_i)$ is the parity of the nth band at the HSPs X $(\mathbf{k} = (\mathrm{a}/\pi, 0, 0))$, Y $(\mathbf{k} = (0, \mathrm{a}/\pi, 0))$, and Z $(\mathbf{k} = (0, 0, \mathrm{a}/\pi))$. For the sonic crystals SC1 and SC2, the sound pressure profiles of the eigenstates at HSPs Γ and X and their corresponding parities are illustrated in Fig. S3~S6, where "+" indicates the even parity while "–" the odd parity. Note that the parities

of Y and Z are the same as X due to the mirror symmetries of the simple cubic lattice. From Eq. S2 we can get $\mathbf{P} = (1/2, 1/2, 1/2)$ for SC1, which indicates a topological phase, while $\mathbf{P} = (0, 0, 0)$ for SC2, denoting a trivial phase. The topology here is protected by the crystalline symmetry of $P_{\mathrm{m\bar{3}m}}$ space group [4].

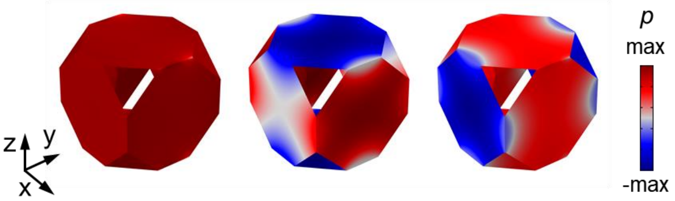

Fig. S3 | Eigenstates at Γ point for bands 1, 2, 3 of SC1. The parities are +, +, +, respectively.

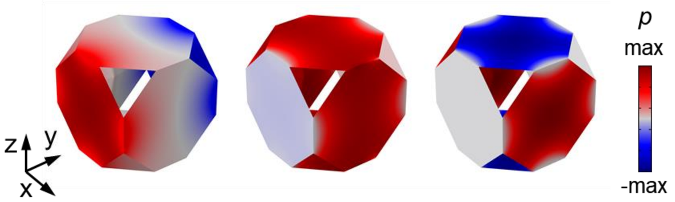

Fig. S4 | Eigenstates at X point for bands 1, 2, 3 of SC1. The parities are +, –, +, respectively.

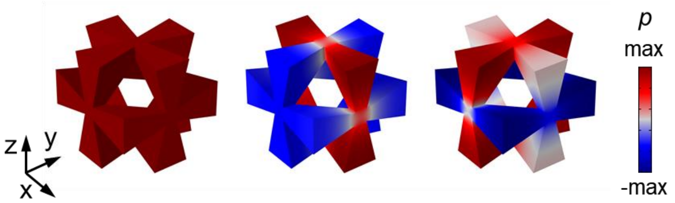

Fig. S5 | Eigenstates at Γ point for bands 1, 2, 3 of SC2. The parities are +, +, +, respectively.

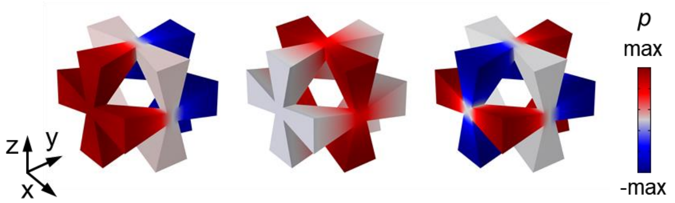

Fig. S6 | Eigenstates at X point for bands 1, 2, 3 of SC2. The parities are –, +, –, respectively.

3. Topological index of interface states

In this work, the interface band inversion occurs with the closing and reopening of the interface band gap, indicating that we can consider only the interface bands to characterize the topological transition. Following Ref. [5], as the supercell with interface bands enjoys C_4

point group symmetry, the topology of the interface band gap can be detected by the following topological index

$$\chi = ([\bar{X}_1^{(2)}], [\bar{M}_1^{(4)}], [\bar{M}_2^{(4)}]), \tag{S5}$$

where $[\Pi_p^{(n)}] = \#\Pi_p^{(n)} - \#\bar{\Gamma}_p^{(n)}$, $\#\Pi_p^{(n)}$ is the number of acoustic interface bands below the interface band gap with C_n symmetry eigenvalue $e^{ip(2\pi/n)}$ at Π point, $p = 0, 1, \ldots, n-1$ and Π stands for $\bar{X}$ and $\bar{M}$.

In our case, however, the first interface state at the $\bar{\Gamma}$ point always merges into and hybridizes with the bulk bands, which leaves the symmetry representations intangible. To solve the problem, we instead turn to the real-space descriptions of the acoustic interface bands and then infer the hidden symmetry representations in momentum space. Firstly, based on the symmetry representations of the acoustic bulk states below the bulk band gap at Γ, X, M and R points, we search the elementary band representations (EBRs) of the space group $P_{m\bar{3}m}$ and conclude the band representations as $(\mathrm{A_{1g}} \uparrow G)_{3d}$ and $(\mathrm{A_{1g}} \uparrow G)_{3c}$ for SC1 and SC2, respectively [6]. In other words, the acoustic bulk states are described by s Wannier orbitals located at three inequivalent Wyckoff positions $3d$ (center of the interface) for SC1 and Wyckoff positions $3c$ (center of hinges) for SC2, as depicted in Figs. S7a and S7b. The three-dimensional bulk polarization $(1/2, 1/2, 1/2)$ of SC1 and $(0, 0, 0)$ of SC2 can also be inferred from the Wannier orbital distribution.

Considering that the supercell with interface states forms an interface of SC1 and SC2, the Wannier orbitals locate at the interface can be easily obtained as $(\mathrm{A} \uparrow G)_{1a} \oplus (\mathrm{A} \uparrow G)_{2c}$ (see Fig. S7c), which are physically related to the interface states. As given in Table S1a, these Wannier orbitals induce the symmetry representations in momentum space as $A_{\bar{\Gamma}} + A_{\bar{X}} + A_{\bar{M}}$ and $(A \oplus B)_{\bar{\Gamma}} + (A \oplus B)_{\bar{X}} + ({}^1E\,{}^2E)_{\bar{M}}$, forming the single interface band and two degenerate interface bands at the $\bar{M}$ point, respectively. Therefore, the interface bands below the interface band gaps for S2 and S3 separately correspond to these two band representations. Based on the character tables of C_2 and C_4 symmetry (see tables S1b and S1c), we can acquire the C_n symmetry eigenvalues of these representations at $\bar{\Gamma}$, $\bar{X}$, and $\bar{M}$ points. Then, the topological indexes of the interface band gaps for S2 and S3 are calculated as $\chi = (0, 0, 0)$ and $\chi = (-1, -1, 1)$, respectively. The different topological indexes imply the topological transition

among the interface states. Furthermore, the two-dimensional polarization $\mathbf{P}$ can also be deduced from the topological index χ, i.e.,

$$P_x = P_y = \frac{1}{2}\left[X_1^{(2)}\right] \ mod\, 1. \tag{S6}$$

Therefore, the polarization $\mathbf{P}$ of the surface band gap is $(0, 0)$ for supercell S2, while a nontrivial quantization of $(1/2, 1/2)$ for S3. The different polarization of two interface band gaps is responsible for the resultant hinge states.

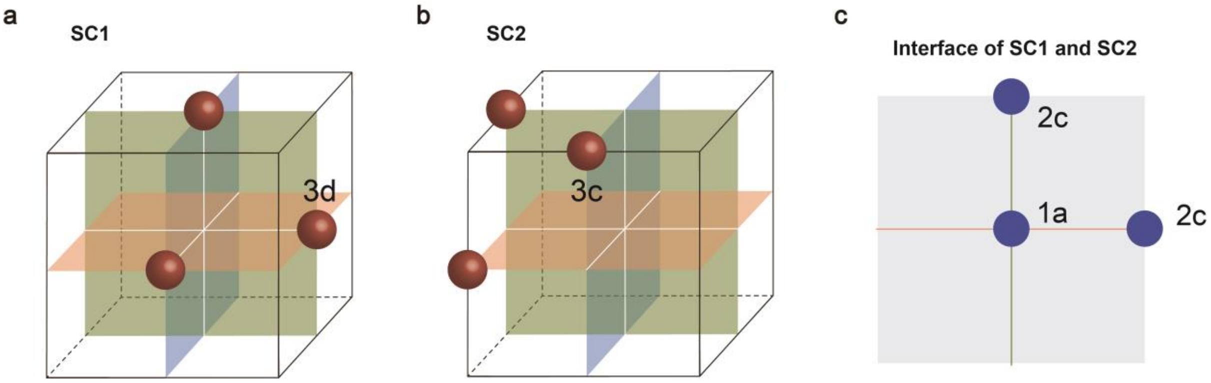

Fig. S7 | Wannier representations of acoustic bulk and interface states. (a) Wannier orbitals locate at the Wyckoff position 3*d* for SC1. (b) Wannier orbitals locate at the Wyckoff position 3*c* for SC2. (c) Wannier orbitals locate at Wyckoff positions 1*a* and 2*c* for the interface states. The red spheres and blue dots denote s orbitals.

a

EBRs	Γ	X	M
$(s)_{1a}$	A	A	A
$(s)_{2c}$	A⊕B	A⊕B	$^1E\,^2E$

b

irreps	E	C_2
A	1	1
B	1	-1

c

irreps	E	C_4^+	C_2	C_4^-
A	1	1	1	1
B	1	-1	1	-1
^1E	1	-i	-1	i
^2E	1	i	-1	-i

Table. S1 | (a) Elementary band representations of $(\mathrm{A} \uparrow G)_{1\mathrm{a}}$ and $(\mathrm{A} \uparrow G)_{2\mathrm{c}}$. (b) and (c) Character tables of C_2 and C_4 point group symmetries, respectively.

4. The $\boldsymbol{k} \cdot \boldsymbol{p}$ theory of the interface Hamiltonian

We use the $\boldsymbol{k} \cdot \boldsymbol{p}$ theory to study the band structure near the interface Dirac-like cone [S5]. The eigenvalue problem in sonic crystals is to solve the following wave equation:

$$-\nabla \cdot \left[{\rho_r}^{-1}(\boldsymbol{r})\nabla p_{n,\boldsymbol{k}_\parallel}(\boldsymbol{r})\right] = \omega_{n,\boldsymbol{k}_\parallel}^2 / v^2 {K_r}^{-1}(\boldsymbol{r}) p_{n,\boldsymbol{k}_\parallel}(\boldsymbol{r}), \tag{S7}$$

where $p_{n,\boldsymbol{k}_\parallel}(\boldsymbol{r})$ is the Bloch wavefunction of the acoustic pressure field with the wavevector

$\boldsymbol{k}_{\parallel} = (k_x, k_y)$ in the nth band, $\omega_{n,\boldsymbol{k}_{\parallel}}$ is the corresponding eigenfrequency. The Bloch function is normalized as $\int_{\text{u.c.}} p^*_{n,\boldsymbol{k}_{\parallel}}(\boldsymbol{r}) \frac{1}{K_r(\boldsymbol{r})} p_{n',\boldsymbol{k}_{\parallel}}(\boldsymbol{r}) = \delta_{nn'}$ with u.c. denoting the unit cell. $\rho_r(\boldsymbol{r}) = \rho(\boldsymbol{r})/\rho_0(\boldsymbol{r})$ and $K_r(\boldsymbol{r}) = K(\boldsymbol{r})/K_0(\boldsymbol{r})$ denote the relative constitutive mass density and bulk modulus, respectively, $v = \sqrt{K_0/\rho_0}$ is the speed of sound in the air host. The Hermitian operator $-\nabla \cdot [\rho_r{}^{-1}(\boldsymbol{r})\nabla]$ can be viewed as the Hamiltonian of the acoustic pressure field.

The key idea of the $\boldsymbol{k} \cdot \boldsymbol{p}$ theory here is using three Bloch wavefunctions $p_{j,\boldsymbol{k_0}}$ ($j = 1, 2, 3$) at the interface Dirac-like point $\boldsymbol{k}_0$ to expand the wavefunctions at $\boldsymbol{k}$ around $\boldsymbol{k}_0$. Recalling that the Bloch wavefunctions have the form $p_{n,\boldsymbol{k}_{\parallel}}(\boldsymbol{r}) = e^{i\boldsymbol{k}_{\parallel}\cdot\boldsymbol{r}} u_{n,\boldsymbol{k}_{\parallel}}(\boldsymbol{r})$ with $u_{n,\boldsymbol{k}_{\parallel}}(\boldsymbol{r})$ being a complete basis set in Hilbert space, we can expand $p_{n,\boldsymbol{k}_{\parallel}}(\boldsymbol{r})$ around $\boldsymbol{k}_0$ as the linear combination of $p_{j,\boldsymbol{k_0}}(\boldsymbol{r})$, i.e.,

$$p_{n,\boldsymbol{k}_{\parallel}}(\boldsymbol{r}) = \Sigma_j A_{n,j}(\boldsymbol{k}_{\parallel}) e^{i(\boldsymbol{k}_{\parallel} - \boldsymbol{k}_0)\cdot\boldsymbol{r}} p_{j,\boldsymbol{k_0}}(\boldsymbol{r}), \tag{S8}$$

where $A_{n,j}$ are expansion coefficients. In principle, the band indices j runs over all bands at $\boldsymbol{k_0}$. In practice, however, we are only interested in three bands in the vicinity of the Dirac-like points, the $\boldsymbol{k} \cdot \boldsymbol{p}$ theory can be restricted to the Hilbert space consisting of only three Bloch wavefunctions. Substituting Eq. S8 into Eq. S7 and utilizing the orthogonality, we obtain the following Hamiltonian:

$$H_{lj}(\Delta \boldsymbol{k}_{\parallel}) = \frac{\delta_{lj}\omega^2_{j,\boldsymbol{k_0}}}{v^2} + \boldsymbol{p}_{lj} \cdot \boldsymbol{q}, \tag{S9}$$

here, $\boldsymbol{q} = \boldsymbol{k}_{\parallel} - \boldsymbol{k_0}$, we have omitted the high-order term of $|\boldsymbol{q}|$. The $\boldsymbol{p}_{lj} \cdot \boldsymbol{q}$ term is analogous to the $\boldsymbol{k} \cdot \boldsymbol{p}$ term in the electronic problem. The matrix element $\boldsymbol{p}_{lj}$ is given by

$$\boldsymbol{p}_{lj} = -i \int_{\text{u.c.}} p^*_{l,\boldsymbol{k}_0}(\boldsymbol{r}) \left\{ \frac{2\nabla p_{j,\boldsymbol{k}_0}(\boldsymbol{r})}{\rho_r(\boldsymbol{r})} + \left[\nabla \frac{1}{\rho_r(\boldsymbol{r})}\right] p_{j,\boldsymbol{k}_0}(\boldsymbol{r}) \right\} d\boldsymbol{r}, \tag{S10}$$

which represents the mode-coupling integrals between degenerate states at the Dirac-like point and determines the linear dispersions around $\boldsymbol{k_0}$. The known is that, $\rho_r(\boldsymbol{r})$ and the operator ∇ are of even and odd parities, respectively, $\boldsymbol{p}_{lj}$ is nonzero only when $p_{l,\boldsymbol{k}_0}(\boldsymbol{r})$ and $p_{j,\boldsymbol{k}_0}(\boldsymbol{r})$ are of opposite parities. The acoustic pressure profiles in Fig. 1d and Fig. 2d in the main text

show that for three states at interface Dirac-like points at both the $\bar{M}$ and $\bar{\Gamma}$ points, there is one s-like state of even parity and two p-like states of odd parity. Furthermore, considering the constraints of the C_4 rotation symmetry, the time-reversal symmetry Θ ($\Theta = K$, K is the complex conjugation), and the mirror symmetries at $\boldsymbol{q} = (q_x, 0)$ and $(0, q_y)$, we reformulate the Hamiltonian on the basis $[s, p_x, p_y]^T$ as

$$H(\boldsymbol{q}) = \begin{bmatrix} \omega_{s,\boldsymbol{k_0}}^2/v^2 & q_x a & q_y b \\ -q_x a & \omega_{p_x,\boldsymbol{k_0}}^2/v^2 & 0 \\ -q_y b & 0 & \omega_{p_y,\boldsymbol{k_0}}^2/v^2 \end{bmatrix}, \tag{S11}$$

where $|a| = |b|$, a and b are pure imaginary numbers. The band dispersion $\omega_{n,\boldsymbol{q}}$ around $\boldsymbol{k_0}$ can be obtained by calculating the following secular equation:

$$\det \begin{vmatrix} \frac{\omega_{n,\boldsymbol{q}}^2 - \omega_{s,\boldsymbol{k_0}}^2}{v^2} & -q_x a & -q_y b \\ q_x a & \frac{\omega_{n,\boldsymbol{q}}^2 - \omega_{p_x,\boldsymbol{k_0}}^2}{v^2} & 0 \\ q_y b & 0 & \frac{\omega_{n,\boldsymbol{q}}^2 - \omega_{p_y,\boldsymbol{k_0}}^2}{v^2} \end{vmatrix} = 0, \tag{S12}$$

which gives two linear bands intersecting with a flat band when $\omega_{s,\boldsymbol{k_0}}^2 = \omega_{p,\boldsymbol{k_0}}^2$ (see Fig. S8), resembling the dispersion in Fig. 2c in the main text.

We then define a mass term $m = \omega_{p,\boldsymbol{k_0}}^2 - \omega_{s,\boldsymbol{k_0}}^2/v^2$, the interface Dirac point is gapped when $m \neq 0$, as shown in Fig. S8. Specifically, the interface band gaps with positive and negative m are topologically distinct. In sonic crystals, m is controlled by the geometric parameters and the topology is characterized by the topological invariant χ when considering the full surface BZ. The topological phase diagram versus "m" is shown in Fig. 2a in the main text.

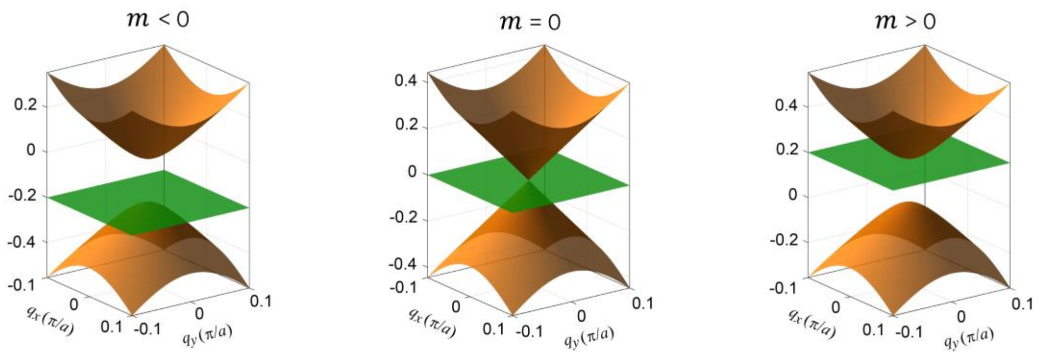

Fig. S8 | $\boldsymbol{k}\cdot\boldsymbol{p}$ interface band structures around $\boldsymbol{q}=0$ with $m>0$, $m=0$, and $m<0$. The Dirac point where two linear bands intersect with a flat band emerges when m vanishes. Band gaps with different topologies are open when $m\neq 0$. The parameters in the $\boldsymbol{k}\cdot\boldsymbol{p}$ Hamiltonian are chosen as $a=b=i$ and $m=\pm 0.2$ (we assume they are dimensionless).

5. Tight-binding model of the interface states

Based on the Wannier description where three single s-wave orbitals are separately occupied at the sites A, B and C (see Fig. S9a), a full Lieb lattice tight-binding (TB) Hamiltonian can be constructed to describe the intriguing physics at the 2D interface. We label three s orbitals as ϕ_{A}, ϕ_{B} and ϕ_{C}, then, the Hamiltonian matrix elements are defined via

$$H_{ij}(\mathbf{R})=\langle\phi_{\mathbf{0}i}H\phi_{\mathbf{R}j}\rangle, \tag{S13}$$

where $\mathbf{R}$ labels the relative lattice vector, i and j denote the sites A, B and C. The matrix element corresponds to a hopping from the orbital ϕ_j in the reference cell to the orbital ϕ_i in the cell $\mathbf{R}$. Using the Fourier transform that $|\chi_j^{\mathbf{k}}\rangle=\sum_{\mathbf{R}}e^{i\mathbf{k}\cdot(\mathbf{R}+\tau_j)}|\phi_{\mathbf{R}j}\rangle$ with τ_j being the relative coordinate vector of the orbitals, we obtain

$$H_{ij}^{\mathbf{k}}=\langle\chi_i^{\mathbf{k}}|H|\chi_j^{\mathbf{k}}\rangle=\sum_{\mathbf{R}}e^{i\mathbf{k}\cdot(\mathbf{R}+\tau_j-\tau_i)}H_{ij}(\mathbf{R}). \tag{S14}$$

We set the couplings between the orbitals at A and B (C) as t_1 (t_2), also, ϵ_{A}, ϵ_{B} and ϵ_{C} as the onsite energy of three orbitals themself. Then, a 3×3 TB Bloch Hamiltonian can be written as

$$H(\mathbf{k})=\begin{pmatrix}\epsilon_{\mathrm{A}} & 2t_1\cos\left(\frac{k_x}{2}\right) & 2t_1\cos\left(\frac{k_y}{2}\right)\\ & \epsilon_{\mathrm{B}} & 2t_2\cos\left(\frac{k_x}{2}-\frac{k_y}{2}\right)+2t_2\cos\left(\frac{k_x}{2}+\frac{k_y}{2}\right)\\ h.c. & & \epsilon_{\mathrm{C}}\end{pmatrix}, \tag{S15}$$

where the lattice constant is set to unity. The corresponding band structures are shown in Figs. S9b-d, as we can see, the difference of the onsite energy at A and B (C) can play the role of the mass term and contribute to the topological phase transition alike to that of the interface states.

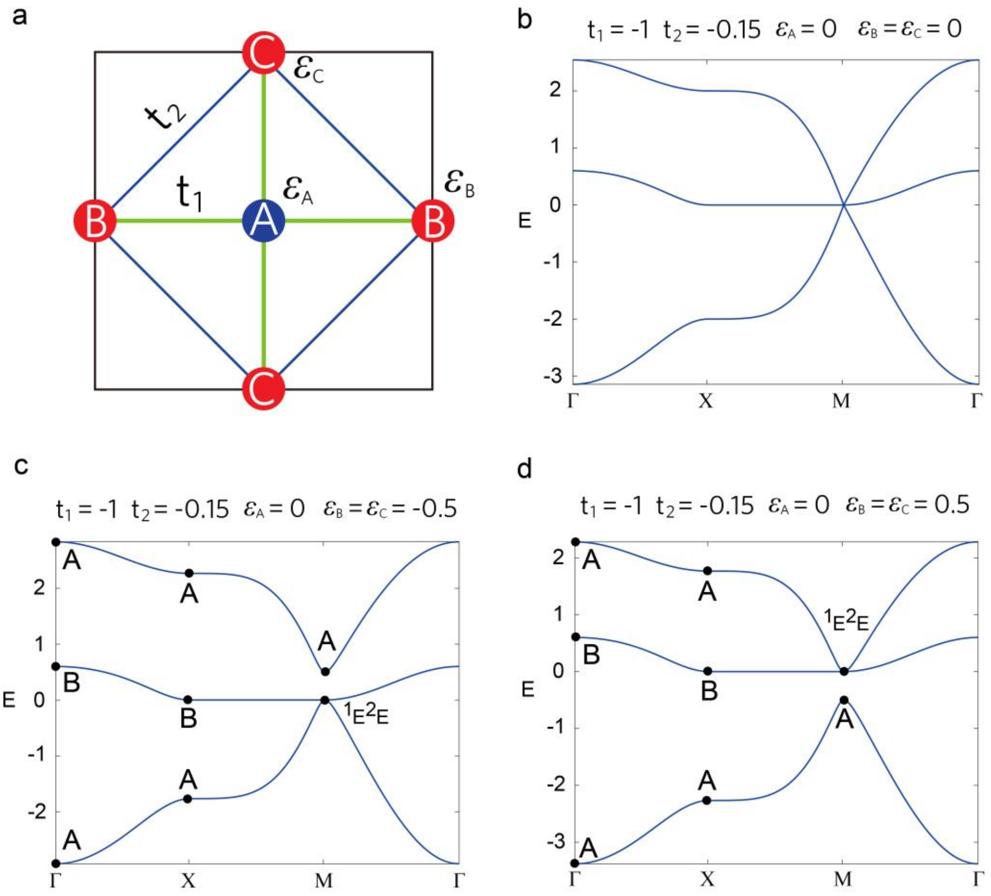

Fig. S9 | Tight-binding model of the interface states. (a) Illustration of the unit cell of the Lieb lattice which captures the physics of the interface. t_1 (t_2) denotes the coupling between the sites A and B (C). ϵ_{A}, ϵ_{B} and ϵ_{C} are onsite energy of three sites. (b)-(d) Band structures of the Lieb lattice with different onsite energy which show the same phase transition as that of the interface states. The symmetry representations at high symmetry points are given to demonstrate the topology. The parameters are given above each figure.

6. Band folding of supercell

When $h_1 = h_2 = 0.27a$ for SC1 and SC2, the resultant supercell is shown in Fig. S10a. For this supercell, the MO mode and the two DI modes accidentally degenerate at the $\bar{M}$ point of the 2D surface BZ. In order to demonstrate the property of the accidental degenerated Dirac-like point, we employ the band folding mechanism and map the Dirac-like point to the $\bar{\Gamma}$ point. The expanded supercell is shown in Fig. S10b. The BZs before and after the band folding are shown in Fig. S10c.

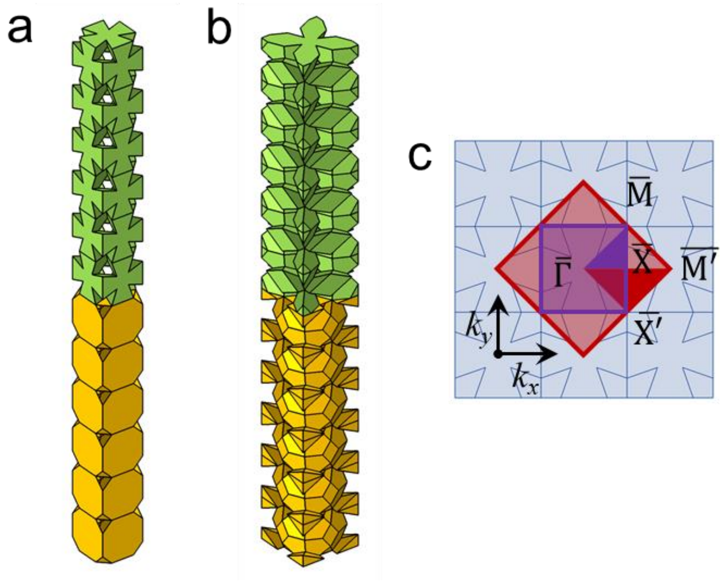

Fig. S10 | Band folding mechanism and the expanded supercell. (a) The supercell when $h_1 = h_2 = 0.27a$. (b) The expanded supercell after band folding. (c) Folding and mapping of BZ. The purple and red areas are the first BZs of the original and expanded supercells, respectively.

7. Gapless topological surface state

The numerically calculated band diagram reveals that the expanded supercell in main text Fig. 2 has gapless topological surface states. To validate that they are gapless, we conduct a simple experiment using the same setup in main text Fig. 3. The acrylic slab with a hole is still glued on the left side of the sample and introduces a point-like sound source. A microphone is located in the middle of the right side of the sample, close to the interface between the trivial and non-trivial sonic crystals. The sound signal in the frequency range 10 kHz to 18 kHz is picked up.

The normalized sound pressure is illustrated in Fig. S11(b). The bulk band gap of the expanded supercell, in the frequency range 12.77 to 15.51 kHz, is illustrated by color grey. It can be seen that remarkable sound propagation is detected in this frequency range, with no noticeable drop or gap. The Dirac-like surface state of the expanded supercell is indeed gapless.

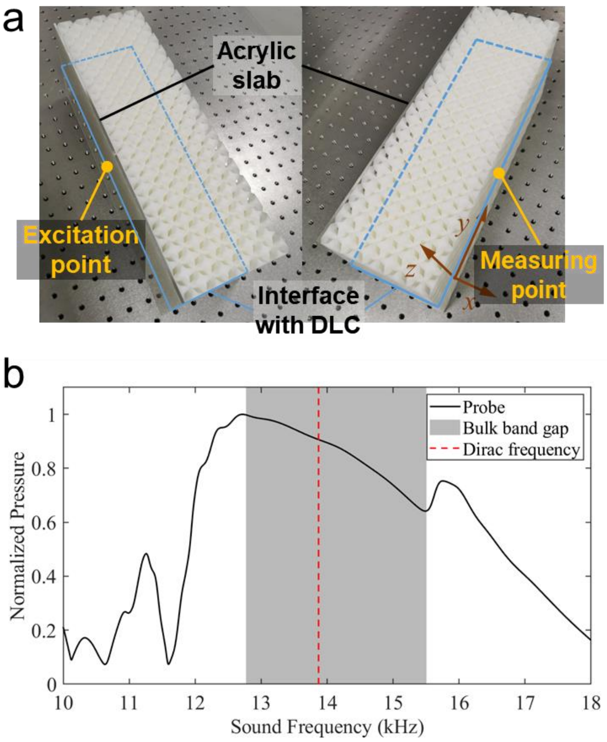

Fig. S11 | Detection of the gapless Dirac-like surface state. (a) Experiment setup. (b) Test result.

8. Effective parameter around the Dirac-like point

We use the effective medium theory to investigate the wave propagation properties of the Dirac-like conical interface states. The interface can be regarded as a 2D material. Using the effective parameter retrieving method from Ref. [7], we obtain the normalized effective density and the inverse of bulk modulus for frequencies around the Dirac-like point, as depicted in Fig. 2e in the main text. Fig. S12 illustrates the supercell for the effective parameter retrieval method. Assuming only one eigenstate is excited when an interface wave impinging on the left of the supercell. The impedance Z of this 2D material is defined as:

$$Z_x = \frac{\langle P_x \rangle}{\langle v_x \rangle}, \tag{S3}$$

where P is the acoustic pressure field on the left face around the interface. v is the acoustic velocity in the direction perpendicular to the left faces. $\langle ... \rangle$ represents the average of P and v. Considering the symmetry of the supercell, we conclude $Z_y = Z_x$.

Assuming the supercell is a uniform 2D medium, the effective mass density ρ and the bulk modulus K can be obtained as:

$$\rho = \frac{k_x \cdot Z_x}{\omega},\ K = \frac{k_x}{\omega \cdot Z_x}, \tag{S4}$$

by varying k_x, we can obtain the relationship between ρ/ρ_0, K_0/K and frequencies around the Dirac-like point.

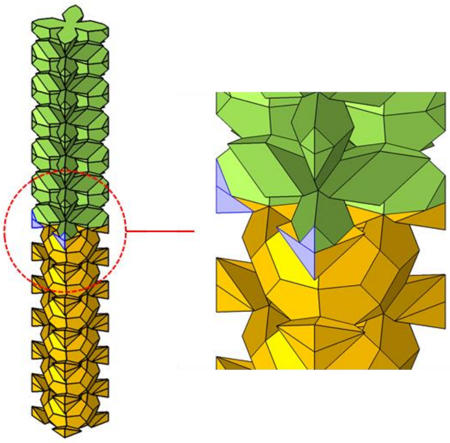

Fig. S12 | Supercell for calculating the effective parameters of the Dirac-like interface states. The pressure field and the velocity field on the blue faces in the vicinity of the interface are considered in the calculation.

9. Robustness of surface states and hinge states

One of the features of topological insulators is that the topological edge states are robust against imperfections. In this research, three mirror symmetries together with the threefold rotation symmetry protect the higher-order topology of the sonic crystals SC1 and SC2. To validate the robustness of the surface states, we introduce symmetry-preserving defects into the ribbon-like supercell shown in Fig. 1d. As shown in Fig. S13a and S13b, the geometry parameter h_1 for SC1 and h_1 for SC2 is increased from 0.175a to 0.35a, respectively. The band structures of the two modified supercells are shown in Fig. S13c and S13d. Since the imperfections are right on the interface between SC1 and SC2, the frequency of surface states changed in a way similar to Fig. 2a — the monopolar states increased in Fig. S13c and decreased in Fig. S13d, while the dipolar states change oppositely. Because of the imperfections, some defect modes (highlighted by the yellow lines) are introduced in the low-frequency region of the bandgap. Overall, although we have introduced significant perturbations, the surface states remain inside the band gap of the bulk, and bulk-surface

correspondence is not broken.

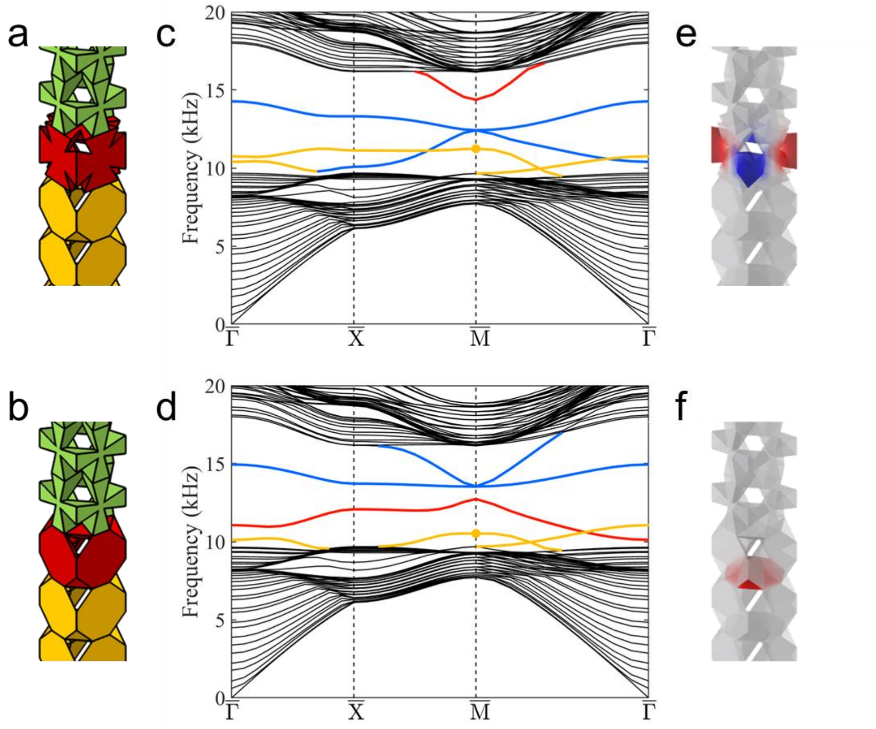

Fig. S13 | Robustness of 2D surface states. (a) and (b) The defective ribbon-like supercell after doubling the value of h_1 for SC1 and SC2. (c) and (d) Band diagram of the defective ribbon-like supercell, where the defect modes are denoted by the yellow lines. (e) and (f) Sound pressure field of the defect modes at $\bar{\mathrm{M}}$ (the yellow dots in the band diagrams).

To validate the robustness of the hinge states, we consider two types of defects. Firstly, we deliberately introduce a substantial number of defects into the hinge supercell. As shown in Fig. S14a, 8 unit cells in the supercell are removed. These defects are only 2*a* away from the hinge. For the defective hinge supercell, the hinge states still exist and the corresponding eigenfrequencies (denoted by the purple dots in Fig. S14e) are almost unaffected. The average relative error of the frequencies is about 0.20%. The sound pressure profile of the defective hinge supercell at $k_y = 0$ is depicted in Fig. S14c. The acoustic wavefunction highly concentrates on the hinge, which is the same as the sound pressure profile in Fig. 4d.

Secondly, we consider defects that appear at where the hinge sits. As shown in Fig. S13b, one of the four unit cells around the hinge is modified. Parameters h_1 for SC2 are increased from 0.175*a* to 0.25*a*. The modified unit cell is highlighted in red color. The hinge states are

calculated and presented in Fig. S14e as the purple squares. It can be seen that the hinge states are still preserved although the eigenfrequencies increase by about 2.5%. The sound pressure profile of the defective hinge supercell at $k_y = 0$ is depicted in Fig. S14d. Similar to Fig. 4d, the acoustic wavefunction still concentrates on the hinge. The numerical results endorse the robustness of the hinge states.

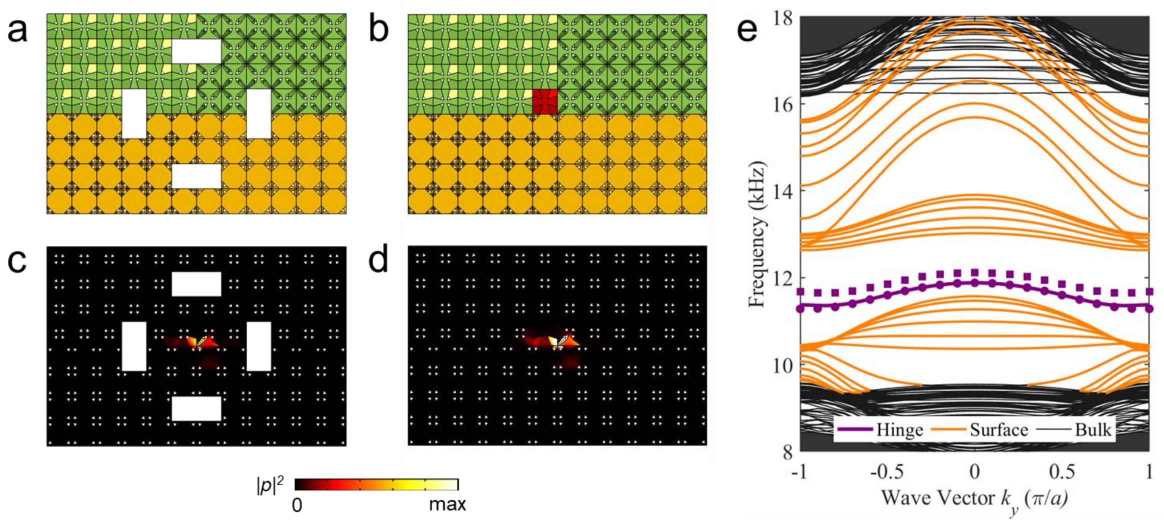

Fig. S14 | Robustness of 1D hinge states. (a) The defective hinge supercell after removing 8 unit cells around the center. (b) The defective hinge supercell after modifying a unit cell of SC2 on the hinge. (c) and (d) Sound pressure field of the hinge state at $k_y = 0$ after introducing defects. (e) Band diagram of the defective hinge supercell, where the hinge states are denoted by the purple line, dots, and squares.

10. Multidimensional propagation of sound in the hinge model

The hinge state in Fig. 4d is within a narrow frequency range 11.37 kHz to 11.88 kHz. Using the experiment in the main text [Fig. 4f], we have visualized the hinge state. By choosing more frequencies, we can investigate the dimensional evolution of sound propagation. The sound pressure field on the scan plane for two other frequencies, 13.30 and 10.80 kHz, are simulated and measured. They are illustrated in Fig. S15 together with the sound profile for 11.40 kHz. When the frequency change from 13.30 to 11.40 and then to 10.80 kHz, the acoustic energy firstly localizes on the 2D topological surface of S2, then localizes on the 1D hinge, and finally change to the 2D topological surface of S3. The experiment results agree well with the

theoretical prediction, visualize the existence of hinge state and the dimensional hierarchy of propagation modes.

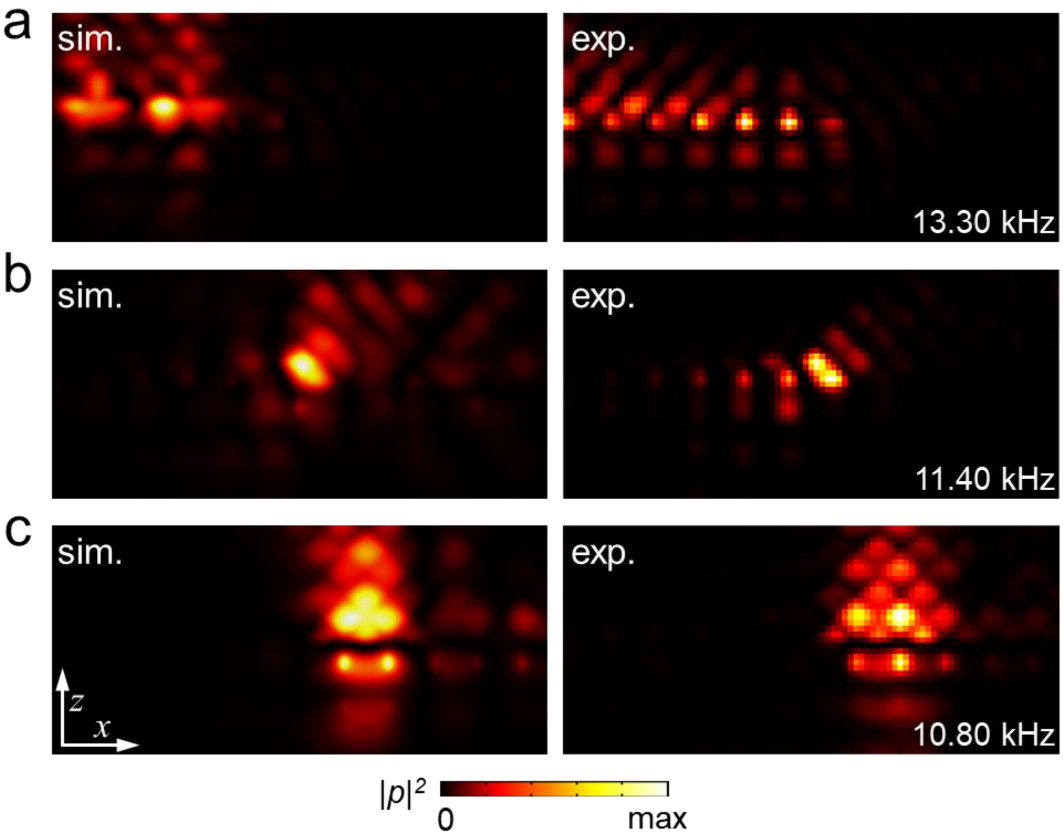

Fig. S15 | Dimensional evolution of sound propagation. (a) (b) (c) The simulated (left) and measured (right) sound pressure field on the scan area for 13.30, 11.40, and 10.80 kHz respectively. With the decreasing of the frequency, the results denote the evolution of the sound transportation from the topological surface states of S2 (13.30 kHz), to the hinge state (11.40 kHz), and then to the topological surface states of S3 (10.80 kHz).

11. Methods

Numerical simulations in this work are conducted by the pressure acoustic module of COMSOL Multiphysics. The density of air is taken as 1.21 kg·m^{-3}, sound speed in air is 343 m·s^{-1}, bulk modulus of air is 1.42×10^{5} Pa. The sample is made of photosensitive resin via stereolithography (SLA), with a fabrication error of 0.1mm. The air-structure interfaces are treated as sound hard boundaries considering acoustically rigid materials construct the sonic crystals. The sound source is a HIVI RT1C-A speaker. The sine wave sound signal is generated

by the built-in sound card of BSWA MC3242 data collector. Sound pressure is picked up by NI 9233 data acquisition card with BSWA MPA416 microphones. The sound profiles are picked up by a microphone fixed on a motorized linear stage. The step length between two scan points is 2 mm.

References:

[1] F. Liu and K. Wakabayashi, Phys. Rev. Lett. **118**, 076803 (2017).

[2] F. Liu, H.-Y. Deng, and K. Wakabayashi, Phys. Rev. B **97**, 035442 (2018).

[3] C. Fang, M. J. Gilbert, and B. A. Bernevig, Phys. Rev. B **86**, 115112 (2012).

[4] X. Zhang, B. Y. Xie, H. F. Wang, X. Xu, Y. Tian, J. H. Jiang, M. H. Lu, and Y. F. Chen, Nat. Commun. **10**, 5331 (2019).

[5] W. A. Benalcazar, T. Li, and T. L. Hughes, Phys. Rev. B **99**, 245151 (2019).

[6] B. Bradlyn, L. Elcoro, J. Cano, M. G. Vergniory, Z. Wang, C. Felser, M. I. Aroyo, and B. A. Bernevig, Nature **547**, 298 (2017).

[7] C. Xu, G. Ma, Z. G. Chen, J. Luo, J. Shi, Y. Lai, and Y. Wu, Phys. Rev. Lett. **124**, 074501 (2020).
